# *Borrelia burgdorferi* spatiotemporal regulation of transcriptional regulator *bosR* and decorin binding protein during murine infection

**DOI:** 10.1038/s41598-020-69212-7

**Published:** 2020-07-27

**Authors:** Elizabeth P. Saputra, Jerome P. Trzeciakowski, Jenny A. Hyde

**Affiliations:** 1 Department of Microbial Pathogenesis and Immunology, College of Medicine, Texas A&M Health Science Center, Bryan, TX USA; 2 Department of Medical Physiology, College of Medicine, Texas A&M Health Science Center, Bryan, TX USA

**Keywords:** Microbiology, Pathogenesis

## Abstract

Lyme disease, caused by *Borrelia burgdorferi*, is an inflammatory multistage infection, consisting of localized, disseminated, and persistent disease stages, impacting several organ systems through poorly defined gene regulation mechanisms. The purpose of this study is to further characterize the spatiotemporal transcriptional regulation of *B. burgdorferi* during mammalian infection of borrelial oxidative stress regulator (*bosR*) and decorin binding protein (*dbpBA*) by utilizing bioluminescent *B. burgdorferi* reporter strains and in vivo imaging. Fluctuating borrelial load was also monitored and used for normalization to evaluate expression levels. *bosR* transcription is driven by two promoters, P_*bb0648*_ and P_*bosR*_, and we focused on the native promoter. *bosR* expression is low relative to the robustly expressed *dbpBA* throughout infection. In distal tissues, *bosR* was the highest in the heart during in the first week whereas *dbpBA* was readily detectable at all time points with each tissue displaying a distinct expression pattern. This data suggests *bosR* may have a role in heart colonization and the induction of *dbpBA* indicates a RpoS independent transcriptional regulation occurring in the mammalian cycle of pathogenesis. These finding demonstrate that *B. burgdorferi* engages unknown genetic mechanisms to uniquely respond to mammalian tissue environments and/or changing host response over time.

## Introduction

Lyme disease, caused by *Borrelia burgdorferi*, is a complex vector-borne infection that causes a multistage inflammatory disease in numerous systems resulting in significant morbidity^1–4^. The multiple stages begin with a localized dermal colonization followed by a dissemination to distal immunoprotective niches, including the central nervous system (CNS), heart, and joints, that can result in the development of neuroborreliosis, Lyme carditis, and/or arthritis, respectively. A robust innate and adaptive immune response is elicited by *B. burgdorferi* that is unable to clear infection or prevent the progression of disease^1^. Antibiotic treatment effectively resolves the disease during early localized infection, but less so after dissemination and late Lyme disease. The Center for Disease Control (CDC) recently reported a substantial increase in the number of cases in the United States from approximately 30,000 to 300,000 per year due to under reporting and less than optimal diagnostic tools^5^. Lyme disease is a significant public health concern with limited treatment options to protect and maintain long term quality of life in humans.

*Borrelia burgdorferi*, a spirochetal bacterium, dynamically regulates gene expression to promote each stage of disease in the vector and mammalian host modulate disease and evade the immune response^2,6–8^. Changes in environmental cues, such as pH, temperature, O_2_, CO_2_, metals and osmotic stress, detected by *B. burgdorferi* result in differential presentation of lipoproteins on the outer membrane to promote adherence, invasion, and colonization^9–17^. Unknown host specific and tissue specific signals induce changes in gene expression that cannot be attributed to known environmental cues defined during in vitro cultivation^18,19^. Expression or repression of mammalian virulence determinants in a temporal and tissue specific manner is essential to progress through the stages of disease. For example, the ectopic constitutive expression of outer surface protein C, *ospC,* that is typically down regulated shortly after infection results in the clearance of the spirochetal pathogen days after inoculation^20^. The specific mechanisms required for borrelial dissemination, distal colonization, and long term infection of tissues are not well understood.

One well characterized mechanism important to establish localized infection is the BosR-Rrp2-RpoN-RpoS pathway that activates gene expression of mammalian virulence determinants in response to an infected tick acquiring a blood meal^21–30^. A complex comprised of the borrelial oxidative stress regulator (BosR), response regulator 2 (Rrp2), and sigma factor (RpoN) interacts with the promoter region of transcriptional activator, *rpoS*. Downstream in this pathway, RpoS activates expression of numerous lipoproteins including outer surface protein C (*ospC*) and decorin binding protein (*dbpBA*)^31,32^. BosR, a metalloregulatory transcriptional regulator, undergoes transcriptional and post-transcriptional regulation in response to environmental signals pH and CO_2_, respectively^14,33^. Murine infection requires *bosR* presumably due its activation of *rpoS* and indirectly the RpoS regulon, but *bosR* expression has not been previously evaluated *in vivo*^22,24^. OspC is required for establishing localized infection and phage display studies suggest it contributes to the colonization of the murine heart^34–37^. Our previous work with bioluminescent reporter *B. burgdorferi* strains to evaluate *ospC* transcript demonstrated unique levels and timing of expression in murine tissues^38^. We observed higher levels of expression in the heart during early infection and an increase of *ospC* in the bladder and joint during late infection. DbpA is also important for establishing a disseminated mammalian infection as it interacts with the extracellular matrices (ECM) through the binding to decorin and type I collagen^39–42^.

We hypothesize that gene regulation is necessary for *B. burgdorferi* to disseminate, colonize unique niches, and/or persist in distal sites during mammalian infection. To address this, we utilized bioluminescent in vivo reporter strains of *B. burgdorferi* to monitor in real time gene expression of *bosR, dbpBA,* and *ospA* during murine experimental infection and in specific tissues. This technology also allows for the evaluation of gene expression independent of variations in borrelial burden in distal colonized tissues. This study demonstrates that *B. burgdorferi* genetic regulation is increasingly complex and newly developed technology allows the dissection of more individualized events. Specifically, we show here that *bosR* is transcriptionally regulated during earlier stages of murine infection with the highest levels observed in the heart. Expression patterns of *dbpBA* are abundant throughout infection and distinct from other RpoS regulated genes, further indicated an independent regulatory mechanism.

## Results

### In vitro characterization of *B. burgdorferi* bioluminescent reporter strains for *bosR, dbpBA*, and *ospA*

To accomplish our goal of evaluating the gene expression patterns of specific genes during murine infection, we generated *B. burgdorferi* bioluminescent reporter strains for *bosR, dbpBA,* and negative control *ospA* similar to our previous studies^38,39^. It is important to note that *bosR* transcription is driven by a promoter immediately upstream (P_*bosR*_) evaluated in this study and the *bb0648* promoter^33^. A borrelial codon optimized firefly luciferase (*luc*) developed in *B. burgdorferi* was utilized to link P_*bosR*_*,* P_*dbp*_*,* and P_*ospA*_ in frame to drive luminescence production as a readout for gene expression^43^. Each reporter was cloned into multicopy borrelial shuttle vector pBBE22 that encodes nicotinamidase restoring mammalian infectivity in a lp25 deficient strain, resulting in pJH488, pJH481, and pJH486, for P_*bosR*_*,* P_*dbp*_*,* and P_*ospA*_, respectively (Table 1)^44^. This bioluminescent shuttle vector is maintained throughout mammalian infection without antibiotic selection due to the selective pressure to maintain the nicotinamidase gene and increases the sensitivity of detection^38,39^. The bioluminescent reporter shuttle vectors were transformed into lp25 deficient *B. burgdorferi*, ML23. The newly generated strains designated ML23 pJH488, ML23 pJH481, and ML23 pJH486 will be referred to as P_*bosR*_*-luc,* P_*dbp*_*-luc,* and P_*ospA*_*-luc* throughout this study for simplification (Table 1).

**Table 1:** Strains and plasmids used in this study.

*B. burgdorferi* strains used in this study		
Strain	Genotype	
ML23	Clonal isolate of strain B31 lacking lp25^45^	
ML23 pJH481	Clonal isolate of strain B31 lacking lp25 containing *bbe22* and *B. burgdorferi* codon optimized *luc* gene under the control of P_*dbp*_ (P_*dbp*_-*luc*)	
ML23 pJH486	Clonal isolate of strain B31 lacking lp25 containing *bbe22* and *B. burgdorferi* codon optimized *luc* gene under the control of P_*ospA*_ (P_*ospA*_-*luc*)	
ML23 pJH488	Clonal isolate of strain B31 lacking lp25 containing *bbe22* and *B. burgdorferi* codon optimized *luc* gene under the control of P_*bosR*_ (P_*bosR*_-*luc*)	
ML23 pBBE22luc	Clonal isolate of strain B31 lacking lp25 containing *bbe22* and *B. burgdorferi* codon optimized *luc* gene under the control of a strong borrelial promoter (P_*flaB*_-*luc*)^39^	

Plasmids used in this study		
Plasmids	Resistance	Description
pCR8/GW/TOPO	spec^R^	ThermoFisher Scientific Gateway PCR cloning/entry vector
pCR2.1	kan^R^	ThermoFisher Scientific PCR cloning/entry vector
pJH434	spec^R^	pCR8/GW/TOPO carrying codon optimized *luc* gene flanked by SalI/NdeI and BamHI sites (promoterless *Bbluc*)
pJH439	spec^R^	*bosR* promoter (P_*bosR*_) engineered with SalI NdeI restriction sites in pCR8/GW/TOPO
pJH435	spec^R^	P_*bosR*_ cloned into pJH434
pJH449	spec^R^	*dbp* promoter (P_*dbp*_) engineered with SalI NdeI restriction sites
pJH472	spec^R^	P_*dbp*_-*luc* cloned into pJH434
pJH454	spec^R^	*ospA* promoter (P_*ospA*_) engineered with SalI NdeI restriction sites
pJH477	spec^R^	P_*ospA*_-*luc* cloned into pJH434
pBBE22	kan^R^	Borrelial shuttle vector encoding *bbe22*^44^
pJH481	kan^R^	pBBE22 carrying P_*dbp*_-*luc*
pJH486	kan^R^	pBBE22 with P_*ospA*_-*luc*
pJH488	kan^R^	pBBE22 encoding P_*bosR*_-*luc*
pβactin	kan^R^	pCR2.1 carrying βactin^46^
precA	kan^R^	pCR2.1 encoding *recA*^47^
p*dbpA*	kan^R^	pCR2.1 with *dbpA*
p*bosR*	kan^R^	pCR2.1 with *bosR*

**Figure 1:**
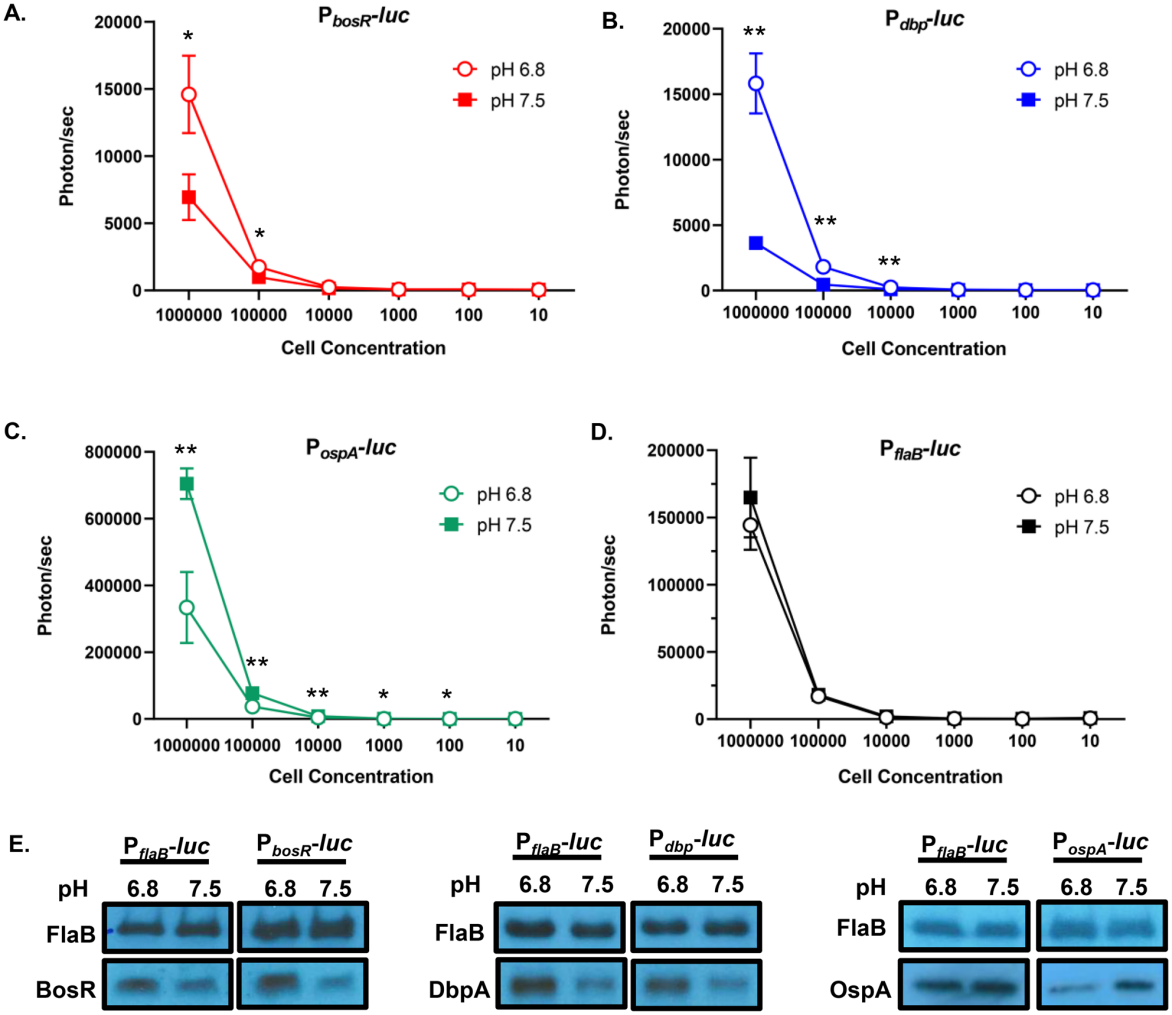
Bioluminescence response of in vitro cultivated *B. burgdorferi* reporter strains to pH. Borrelial strains (**A**) P_*bosR*_*-luc*, (**B**) P_*dbp*_*-luc*, (**C**) P_*ospA*_*-luc*, (**D**) P_*flaB*_*-luc* were grown in triplicate to mid-log phase and serially diluted from 10^6^ to 10 cells. *B. burgdorferi* strains were treated with D-luciferin. Luminescence was measured for each sample after subtracting the background levels and averaged. The error bars in the graphs represent standard error. Statistical significance was determined by *t*-test (*p < 0.05 and **p < 0.01). (**E**) Cell lysates from P_*bosR*_*-luc,* P_*dbp*_*-luc*, P_*ospA*_*-luc*, and P_*flaB*_*-luc* were immunoblotted and probed with anti-sera to antigen indicated on the left. Constitutively produced borrelial FlaB was used as a control for cell equivalents between samples.

Our first step was to ensure the borrelial bioluminescent reporters were able to respond to environmental cues known to alter *bosR*, *dbpBA*, and *ospA* transcripts. Reporter strains were grown under pH 6.8 and 7.5 to mimic mammalian and tick conditions for in vitro luminescence assays and Western analysis (Fig. 1). Mammalian virulence determinants reporters P_*bosR*_*-luc* and P_*dbp*_*-luc* are transcriptionally induced at pH 6.8 relative to pH 7.5, as expected (Fig. 1A, B)^33,48^. Western analysis demonstrated an increase of BosR and DbpA in P_*bosR*_*-luc* and P_*dbp*_*-luc* strains, respectively, at pH 6.8 when compared to pH 7.5 (Fig. 1E). FlaB production was unchanged under the different pH conditions and used as an equivalent loading control for Western analysis. OspA an important lipoprotein for tick midgut colonization, therefore P_*ospA*_*-luc* is a useful negative control during mammalian infection^49,50^. P_*ospA*_*-luc* demonstrated elevated bioluminescence and OspA production at pH 7.5 as expected (Fig. 1C, E)^48^. The previously reported constitutive luminescent *B. burgdorferi* P_*flaB*_*-luc* was used as a negative control for a transcript not altered by environmental changes in vitro and a readout for bacterial load in vivo (Fig. 1D)^38,39^. Together, these results indicate the reporter strains appropriately respond to environmental cues to represent the transcriptional regulation of *bosR, dbpBA*, and *ospA*.

**Figure 2:**
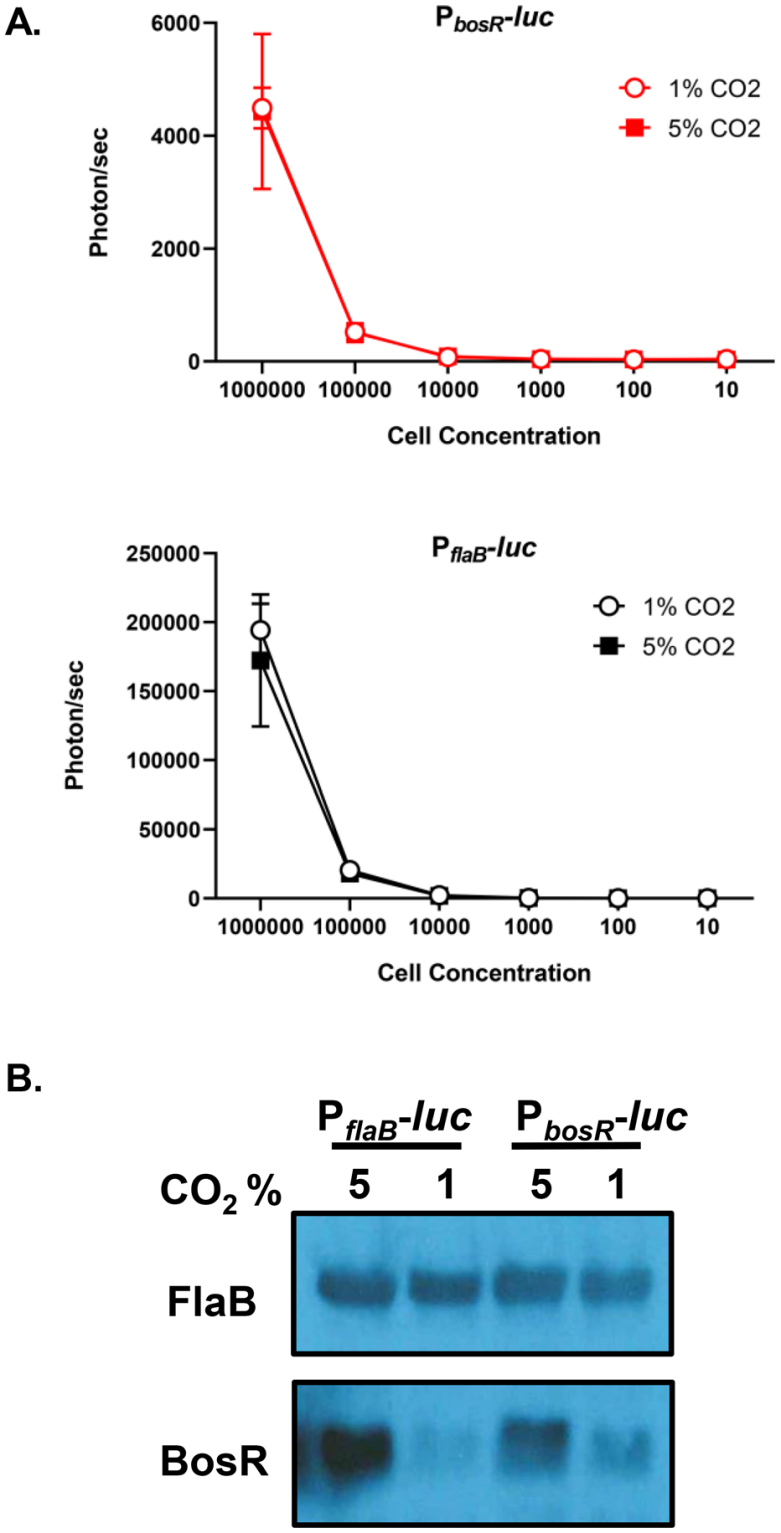
Luminescence of *B. burgdorferi* P_*bosR*_*-luc* reporter strain to CO_2_. Triplicate borrelial strains, P_*flaB*_-luc and P_*bosR*_-luc, were grown in 1% or 5% CO_2_ to mid-log phase and samples were harvested for bioluminescence assay (**A**) or Western analysis (**B**). (**A**) Cells were serially diluted from 10^6^ to 10 cells, treated with D-luciferin, and measured for bioluminescence. Samples were averaged and *t*-test analysis determined statistical significance. Error bars represent standard error. P_*flaB*_-luc and P_*bosR*_-luc demonstrate no difference in bioluminescence with changes in CO_2_ levels, as expected. (**B**) Samples were immunoblotted and probed with anti-sera to antigen indicated on the left. Constitutively produced borrelial FlaB was used as a control for cell equivalents between samples and are not impacted changes in cultivation conditions.

As with other bacterial pathogens, *B. burgdorferi* undergoes post-transcriptional regulation that is not addressed with this methodology, which is relevant for BosR that is transcriptionally and post-transcriptionally regulated^14,33^. P_*bosR*_*-luc* was grown in media equilibrated under 1% and 5% CO_2_ atmospheric conditions resulting in no difference in luminescent emission, but an increase in BosR production at elevated CO_2_ (Fig. 2). As in the previous experiment, P_*flaB*_*-luc* is a bioluminescent assay negative control for environmental changes and FlaB is a Western blot equivalent loading control. This study focused on a single step of genetic regulation of *B. burgdorferi,* specifically for *bosR* and *dbpBA* transcription*,* during murine infection.

### Temporal evaluation of *B. burgdorferi* reporter strains during murine infection

**Figure 3:**
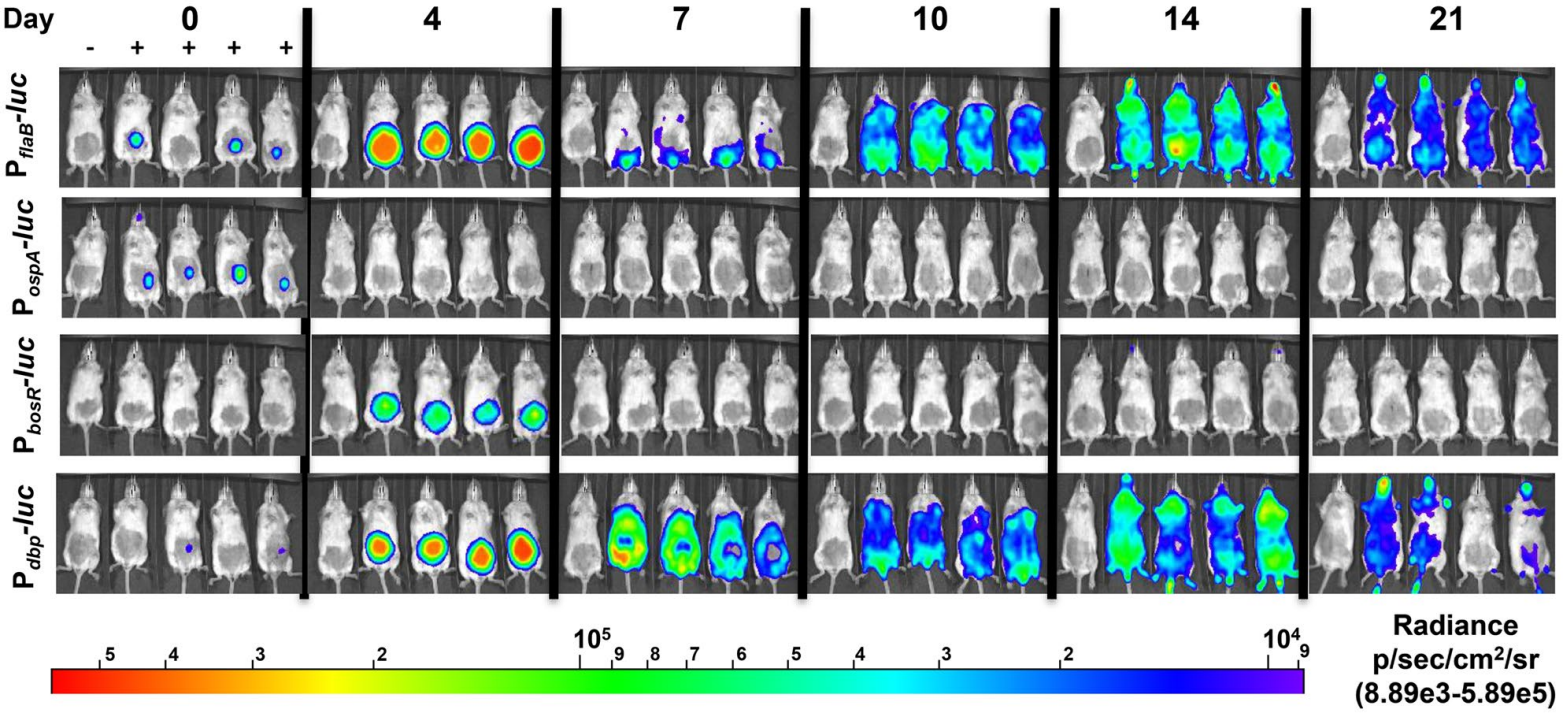
Temporal expression *B. burgdorferi* reporter strains during experimental murine infection. The expression of *bosR*, *dbpBA*, and *ospA* during murine infection was evaluated over time. Bioluminescent *B. burgdorferi* P_*flaB*_*-luc*, P_*bosR*_*-luc,* P_*dbp*_*-luc,* and P_*ospA*_*-luc* infected Balb/c mice by intradermal injection with a 10^5^ inoculum dose. P_*flaB*_*-luc* serves as a monitor of bacterial load. D-luciferin treatment, represented by + , was administered 1 h and 4, 7, 10, 14, and 21 days post-infection for in vivo imaging. A background control mouse (far left in each image and indicated by −) is included in each group that is not treated with D-luciferin. Exposures of 10 min was captured for images. Normalization of images was performed to remove all visible background bioluminescence from the no luciferin treated mouse across all strains and time points. All images from each time point and strain were normalized to the same radiance range of 8.89 × 10^3^ to 5.89 × 10^5^.

**Figure 4:**
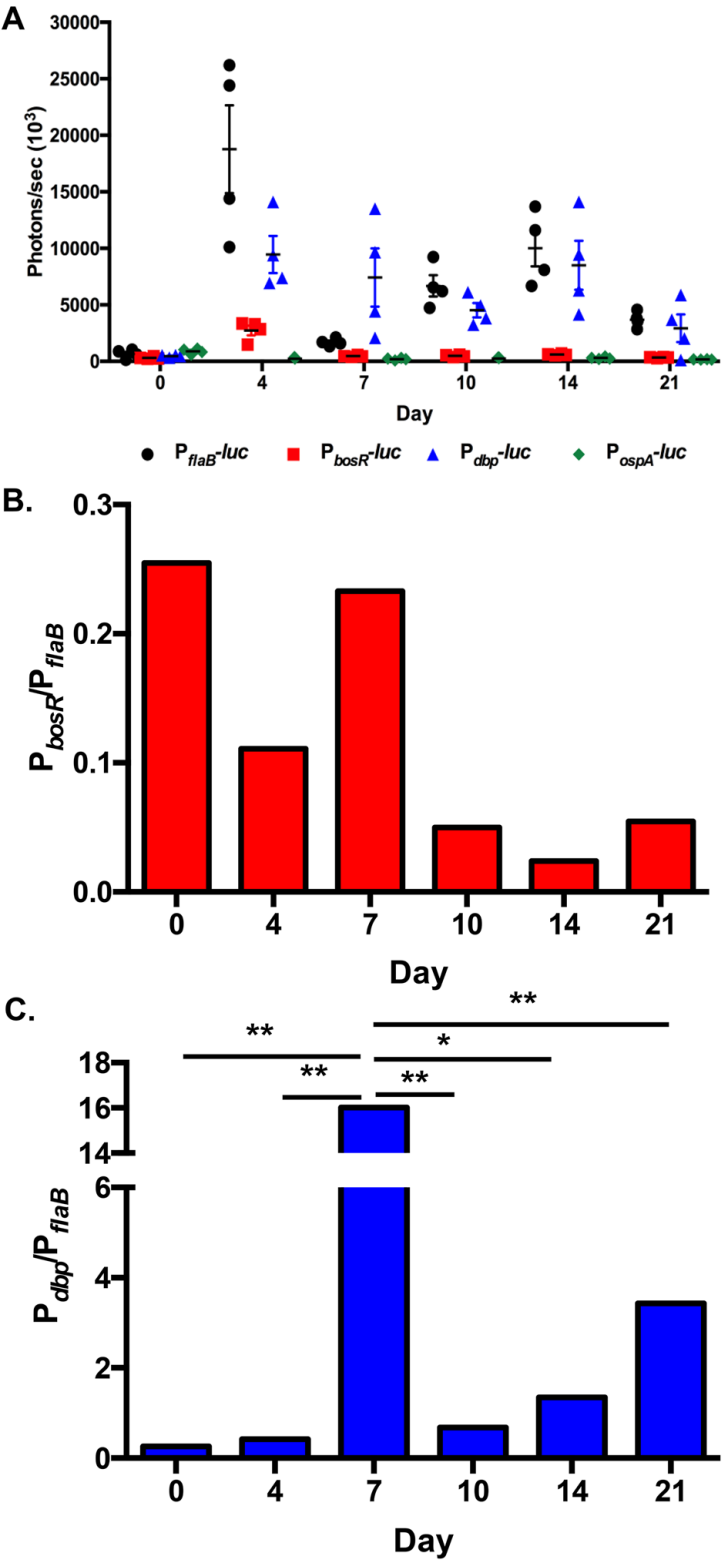
Quantitation of in vivo bioluminescence of *B. burgdorferi* reporter strains. (**A**) Exposures with counts range from 600 to 60,000 were used for quantitation of bioluminescence. One-way ANOVA showed significant difference in bioluminescence over time for P_*flaB*_*-luc* (*p* < 0.0001*),* P_*bosR*_*-luc* (*p* < 0.0001)*,* P_*dbp*_*-luc* (*p* < 0.0068)*,* and P_*ospA*_*-* (*p* < 0.0001). (**B**) P_*bosR*_*-luc* normalized for changes in borrelial load (P_*flaB*_*-luc*)*.* Permutation analysis indicated no significant differences of P_*bosR*_*-luc/*P_*flaB*_*-luc* between time points. (**C**) P_*dbp*_*-luc* normalized for changes in borrelial load with P_*flaB*_*-luc.* Permutation analysis was used to determine the significant difference between time points for each reporter strain. P_*dbp*_*-luc/* P_*flaB*_*-luc* on day 7 was significantly different relative to other time points with a *p* value range of 0.0016 to 0.0129. * represents *p* < 0.05 and ** designates *p* < 0.01.

An in vivo bioluminescent reporter infectivity study was performed to characterize the dynamic regulation of *bosR, dbpBA,* and *ospA* during mammalian infection. *B. burgdorferi* P_*flaB*_*-luc*, P_*bosR*_*-luc,* P_*dbp*_*-luc,* or P_*ospA*_*-luc* were intradermally (ID) inoculated in Balb/c mice that were monitored by in vivo imaging for the emission of light. The constitutively expressed P_*flaB*_*-luc* is a control for fluctuations in borrelial burden over time and in different colonization sites that is used for normalization allowing for the analysis of changes in transcription only. Bioluminescent imaging, represented as photons/sec or radiance, are normalized for background utilizing an infected mouse not treated with D-luciferin in each group. As observed in previous bioluminescent studies with constitutive P_*flaB*_*-luc* a strong localized colonization develops at the site of inoculation followed by the dissemination of the bioluminescent signal to distal tissues and throughout the murine skin with bacterial load fluctuating throughout the 21 day time course (Figs. 3, 4A)^38,39^. At the brief 2 h time point bioluminescent levels are representative of *B. burgdorferi* response to in vitro microaerophilic growth conditions as indicated by the robust emission from P_*ospA*_*-luc* infected mice (Fig. 3). *ospA* is one of the highest expressed genes during cultivation of *B. burgdorferi*^48^. P_*ospA*_*-luc* bioluminescence is observed and measurable 2 h after ID injection, but quickly declines to background levels at all other time points (Figs. 3, 4A). The multicopy P_*ospA*_*-luc* reporter is down regulated in the murine dermis within 24 h post-infection (data not shown) and does not return in the same manner as native *ospA*^51,52^. This demonstrates the in vivo bioluminescence *B. burgdorferi* reporter system can accurately represent bacterial burden and transcriptional changes, both positively and negatively, in the murine model.

We assessed the temporal transcriptional regulation of *bosR* during mammalian infection. Expression of *bosR* is low under in vitro microaerophilic condition as represented by the level of emission from P_*bosR*_*-luc* infected mice 2 h post infection (Fig. 3). P_*bosR*_*-luc* bioluminescence peaks with 4.3 × 10^5^ photons/s (p/s) at 4 days post infection (dpi) followed by a 11.5-fold and 22-fold reduction at 7 and 21 dpi, respectively (Figs. 3, 4A). One-way ANOVA (*p* < 0.0001) indicates a significant difference in P_*bosR*_*-*luc bioluminescence during the course of a 21 day infection. Normalization of P_*bosR*_*-luc* for changes in borrelial burden, represented as P_*bosR*_*-*luc/P_flaB_*-luc*, indicates the highest expression of *bosR* is during the first 7 days of infection suggesting this transcriptional regulator is important for early stages of pathogenesis and establishing infection in the murine model (Fig. 4B). Permutation analyses of P_*bosR*_*-*luc/P_flaB_*-luc* did not show a significant difference when comparing time points likely due to low level signal and variability of bioluminescent emission between mice. The P_*bosR*_*-luc *in vivo expression pattern is similar to our previous study with P_*ospC*_*-luc*, as one would expect, due to the role of BosR in the transcriptional activation of *rpoS* in complex with Rrp2 and RpoN^22–25^.

The RpoS regulation of *dbpBA* in response to environmental signals under in vitro cultivation conditions is well understood, while regulation of this important lipoprotein in physiologic environment of murine infection is less clear^10,11,31,48^. Expression of *dbpBA* during in vitro cultivation is substantially lower than *flaB* or *ospA* and is also observed in Balb/c mice intradermally inoculated for 2 h with P_*dbp*_*-luc* (Figs. 1, 3, 4A). *B. burgdorferi* P_*dbp*_*-luc* adapts to the murine host resulting in a dramatic increase in bioluminescence beginning at 4 dpi and continuing through 21 dpi (Figs. 3, 4A). One-way ANOVA analysis of P_*dbp*_*-luc* photon/sec resulted in a *p*-value of 0.0068 indicating bioluminescence significantly changes over time. The bioluminescent emission from P_*dbp*_*-luc* exceeds constitutive P_*flaB*_*-luc* at 7, 14, and 21 dpi indicating that *B. burgdorferi* robustly expresses this specific RpoS regulated lipoprotein during mammalian infection. Normalizing for bacterial load, as shown by P_*dbp*_*-luc*/P_*flaB*_*-luc*, *dbpBA* expression increases 15.6 fold from 4 to 7 dpi that was followed by a 15.3 fold decline at 10 dpi (Fig. 4C). Bioluminescence increased fivefold from the low emission at day 10 to the second peak at 21 dpi. Overall the data suggests that high levels of *dbpBA* expression may contribute to borrelial pathogenesis during dissemination and maintain colonization of distal tissues (Fig. 4C). P_*dbp*_*-luc*/P_*flaB*_*-luc* bioluminescence at 7 dpi is significantly different by permutation analysis in comparison to all time points with *p* values ranging from 0.0457 to 0.01032. The lack of significance between time points indicates the maintained robust expression of *dbpBA*. The bioluminescent emission of P_*dbp*_*-luc B. burgdorferi* has a distinct in vivo temporal expression pattern compared to P_*bosR*_*-luc* or previously published P_*ospC*_*-luc*, which peaked at 7 dpi followed by a decline in bioluminescence that rises again at day 21 (Figs. 3, 4A)^38^. The difference in P_*ospC*_*-luc* and P_*dbp*_*-luc* during murine infection is observed in the overall intensity and temporal production of bioluminescent emission when evaluating the whole mouse.

**Figure 5:**
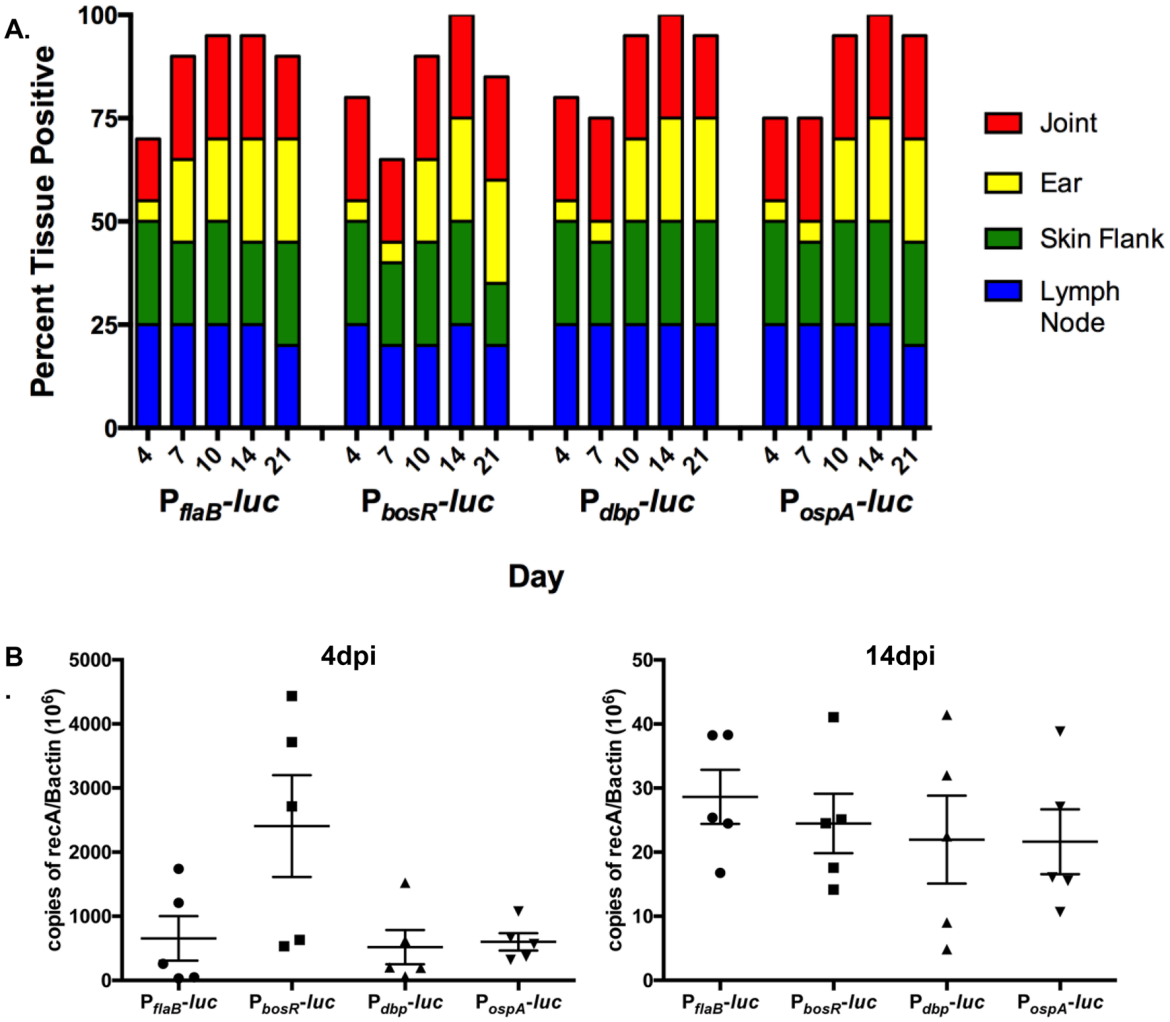
Bacterial burden of *B. burgdorferi* bioluminescent reporter strain infected mice. (**A**) Qualitative kinetics of infection with *B. burgdorferi* reporter strains in Balb/c mice. Upon completion of optical imaging for tissues from Balb/c mice infected with 10^5^ P_*flaB*_*-luc*, P_*bosR*_*-luc,* P_*dbp*_*-luc,* and P_*ospA*_*-luc*, the animals were sacrificed and the skin flank, ear, inguinal lymph node and tibiotarsal joint were harvested on day 4, 7, 10, 14 and 21 for in vitro cultivation to monitor the outgrowth of *B. burgdorferi.* The x-axis indicates the strains and time points tested (in days). The y-axis displays the total percentage of culture positive tissues for a given time point comprised of the five tissues per strain. Groups of mice were equivalently infected with each reporter strain. (**B**) Bacterial burden equivalent between groups of mice infected with *B. burgdorferi* reporter strains. Flank skin samples of Balb/c mice infected with 10^5^ P_*flaB*_*-luc*, P_*bosR*_*-luc,* P_*dbp*_*-luc,* and P_*ospA*_*-luc* were harvested on 4 and 14 dpi for qPCR analysis of borrelial genomes (*recA*) per copies of 10^6^ β-actin. Error bars represent standard error. Statistical analysis using the Mann–Whitney test indicated a lack of significance between all strains at 4 and 14 dpi.

To verify mice were infected with equivalent numbers of *B. burgdorferi* P_*flaB*_*-luc,* P_*bosR*_*-luc,* P_*dbp*_*-luc,* or P_*ospA*_*-luc* harvested tissues underwent qualitative and quantitative evaluation (Fig. 5). At each day of imaging the inguinal lymph node, skin flank, ear, and the tibiotarsal joint were collected and transferred to complete BSK to monitor the outgrowth of viable *B. burgdorferi* (Fig. 5A). Tissues were qualitatively scored positive once motile borrelial cells were observed by dark field microscopy. All tissues have similar colonization of *B. burgdorferi* between all strains at each time point indicating groups of mice were inoculated with approximately the same number of cells (Fig. 5A). DNA was isolated from skin flank samples harvested at 4 and 14 dpi to determine the number of borrelial genomic copies (*recA*) per 10^6^ mouse β-actin (Fig. 5B). One-way ANOVA and individual Mann–Whitney analysis indicated there was not a significant difference in the borrelial burden of P_*flaB*_*-luc,* P_*bosR*_*-luc,* P_*dbp*_*-luc,* or P_*ospA*_*-luc* infected skin flanks verifying mice were equivalently infected (Fig. 5B).

### *bosR* expression in murine tissues

**Figure 6:**
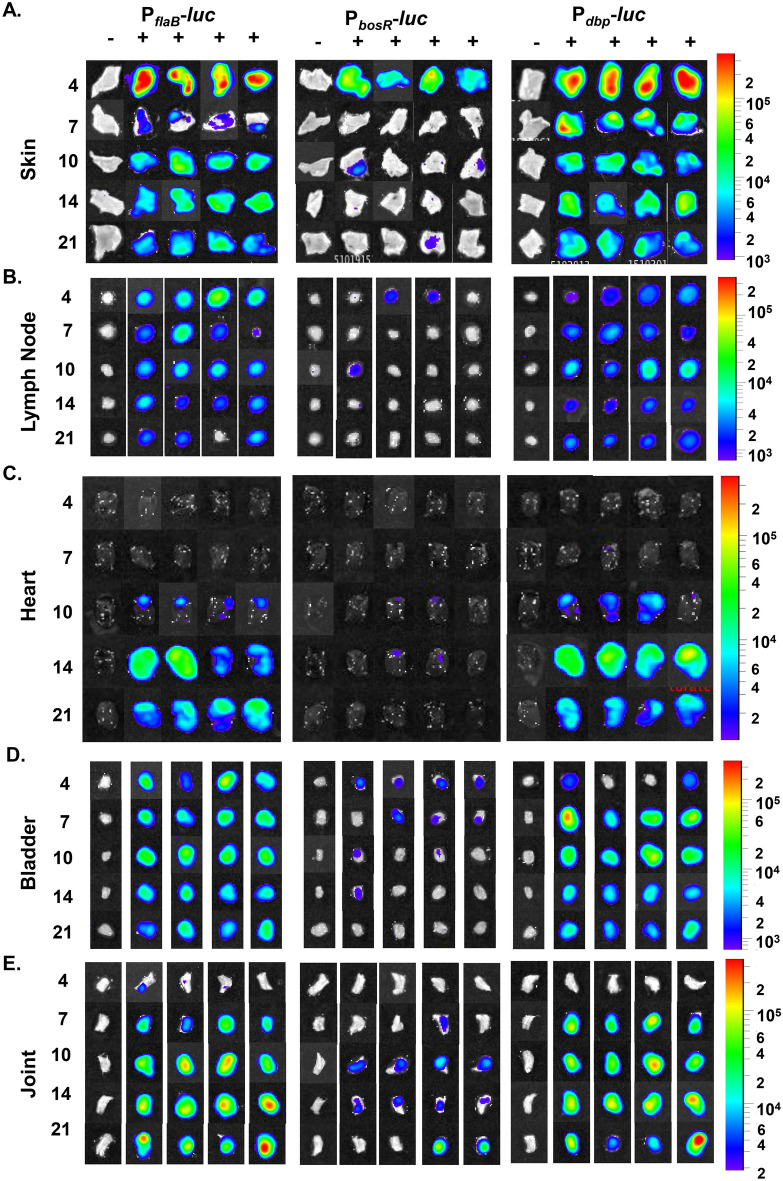
Temporal and spatial expression of *B. burgdorferi* containing P_*flaB*_*-luc*, P_*bosR*_*-luc,* and P_*dbp*_*-luc* in murine tissues. Individual tissues from Balb/c mice infected with from P_*flaB*_*-luc*, P_*bosR*_*-luc,* and P_*dbp*_*-luc B. burgdorferi* were quantitatively evaluated for bioluminescence at 4, 7, 10, 14, and 21 dpi. P_*flaB*_*-luc* represents borrelial load. P_*bosR*_*-luc* and P_*dbp*_*-luc* represents transcription of *bosR* and *dbp,* respectively. Tissues aligned under the (−) were not treated with D-luciferin serving as a background control and (+) designated tissues were harvested from mice treated with a double bolus of D-luciferin prior to sacrifice. Images of individual tissues are from 10 min exposures and normalized to the same radiance range (on the right) across all borrelial strains for each tissue. (**A**) Underside of flank skin (9.1e3–3.7e5 radiance), (**B**) inguinal lymph node (7.05e2–3.7e5 radiance), (**C**) heart (1.11e3-3.7e5 radiance), (**D**) BLADDER (6.9e2–3.7e5 radiance), (**E**) tibiotarsal joint (1.93e3–3.62e5 radiance).

Transcriptional regulator *bosR* is regulated by environmental signal, temperature, pH, O_2_ and CO_2_, and is essential for murine infection^9,14,33^. *B. burgdorferi* differentially regulates gene expression in a tissue specific manner over the course of infection that is not replicated under environmental in vitro conditions^2,18,38,53^. The tissue specific transcriptional regulation of *bosR* during murine infection is unknown. Ex vivo imaging of individual murine tissues infected with bioluminescent *B. burgdorferi* allows for the direct spatiotemporal analysis of gene regulation while taking into account changes in borrelial burden. We harvested tissues, including skin flank, inguinal lymph node, heart, bladder, and tibiotarsal joints, from mice infected with bioluminescent *B. burgdorferi,* P_*flaB*_*-luc,* P_*bosR*_*-luc,* and P_*dbp*_*-luc* reporters*,* to quantitate radiance emission at each time point beginning at 4 dpi (Figs. 6, S1). Similar to in vivo imaging, mice were treated with a double bolus of D-luciferin with the exception of 1 mouse that served as the background control for normalization.

**Figure 7:**
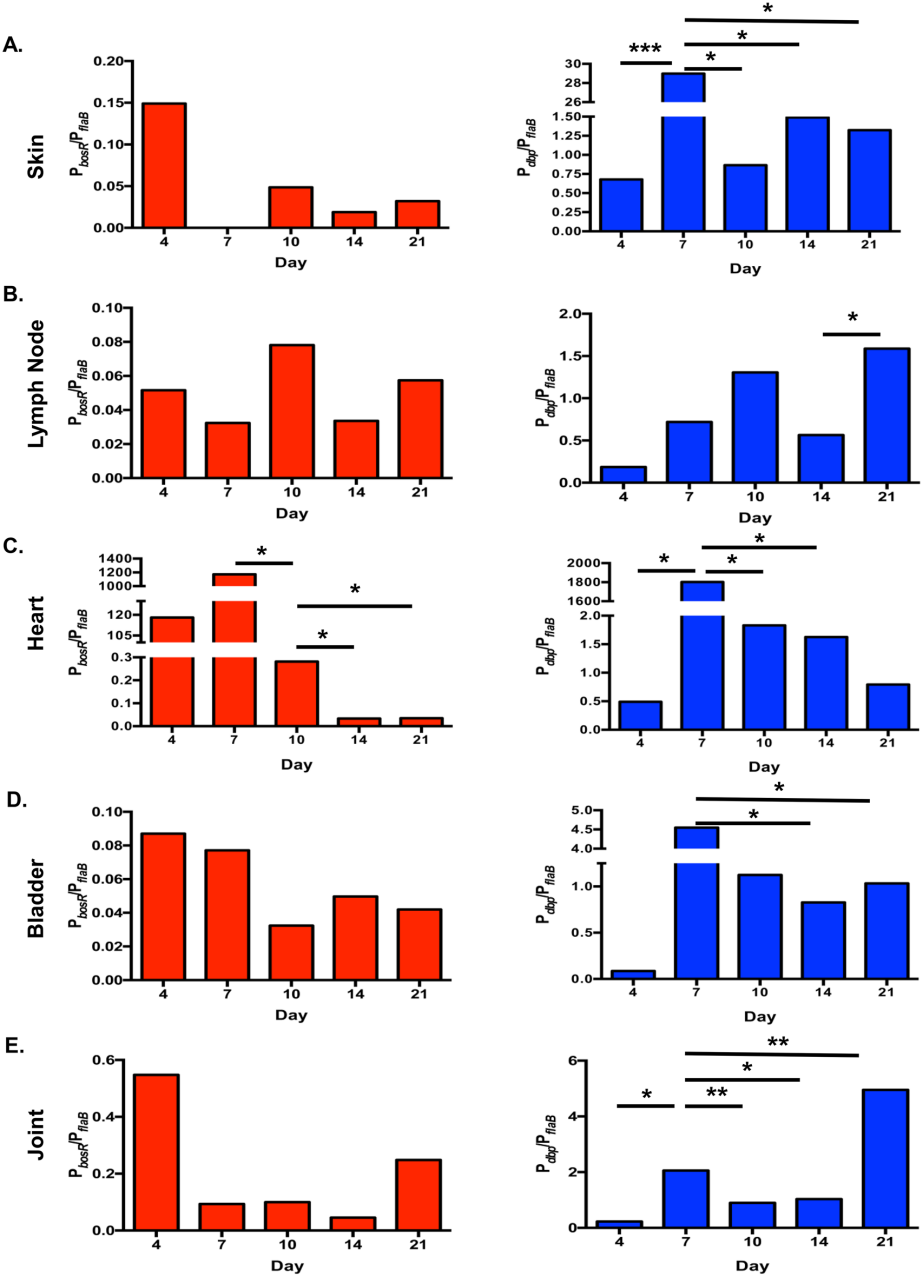
Tissue specific regulation of *bosR* and *dbpBA* expression independent of fluctuation in *B. burgdorferi* burden. To account for changes in bacterial load during the course of infection P_*bosR*_*-luc* infected tissues were normalize to P_*flaB*_*-luc* resulting in the ratio P_*bosR*_/P_*flaB*_. (**A**) Underside of skin flank, (**B**) inguinal lymph node, (**C**) heart, (**D**) bladder, (**E**) tibiotarsal joint. Permutation analyses was perform to randomly compare all possible ratios and determine statistical significance with * representing *p* < 0.05, **p < 0.01, and ***p < 0.001.

P_*flaB*_*-luc* infected tissues were measured for the changes in bioluminescence at each site and time point to represent borrelial burden and serve as a normalization control in this study (Figs. 6, S1). Our previous work demonstrated a strong correlation between P_*flaB*_*-luc* bioluminescence emission and borrelial load^39^. P_*flaB*_*-luc* infected skin, heart, and joint showed statistically significant differences over time with a *p* values of < 0.0001, 0.0168, and 0.0007, respectively, by one-way ANOVA (Fig. S1). The inguinal lymph node and bladder did not significantly fluctuate over time and were infected at consistent levels throughout infection. Overall, each examined tissue displayed a distinct P_*flaB*_*-luc* bioluminescence signal indicating the unique microenvironment of each site alters the number of *B. burgdorferi* at a given time. The radiance from P_*flaB*_*-luc* infected tissues were used to normalize bioluminescence from P_*bosR*_*-luc* infected mice to account for differing borrelial burden (Fig. 7).

We evaluated the spatiotemporal expression of transcriptional regulator *bosR* during infection in P_*bosR*_*-luc* inoculated mice (Fig. 6). Radiance (p/sec/cm^2^/sr) was measured from tissues of four D-luciferin treated P_*bosR*_*-luc* infected mice and normalized for background to one untreated infected mouse (Figs. 6, S1). Over the course of infection bioluminescent emission significantly changed when analyzed by one-way ANOVA in the skin flank, heart, and bladder with *p-*values of 0.0028, 0.00035, and 0.0155, respectively. Bioluminescent emission in the lymph node was low and highly variable during murine infection indicating *bosR* was not steadily induced or necessary in this particular tissue in a manner previously observed with P_*ospC*_*-luc* (Figs. 6, S1B)^38^. The joint was consistently infected as observed in IVIS images, thus did not significantly differ when comparing time points (Figs. 6, S1E). The ratio P_*bosR*_*-luc*/P_*flaB*_*-luc* represents *bosR* expression normalized for changes in borrelial burden (Fig. 7). The temporal expression of *bosR* is very similar to *ospC* in several tissues^39^. Specifically, P_*bosR*_*-luc*/P_*flaB*_*-luc* have higher ratios at earlier time points in the skin flank and heart (Fig. 7A, C). Expression in the bladder steadily declines over time (Fig. 7D). In the joint, ratios peak at 4 and 21 dpi as observed with P_*ospC*_*-luc*/P_*flaB*_*-luc* (Fig. 7E). Permutation analysis of P_*bosR*_*-luc*/P_*flaB*_*-luc* ratios was performed and only the heart was significantly different between 7 versus 10 dpi, 10 versus 14 dpi, and 10 versus 21 dpi with *p*-values of 0.0123, 0.0111, and 0.0111, respectively (Fig. 7C). Together, this data indicates that *bosR* has moderate transcriptional regulation during murine infection over time in a tissue specific manner. It is likely that post-transcriptional regulation of *bosR* has a greater impact on the RpoS pathway and murine pathogenesis.

**Figure 8:**
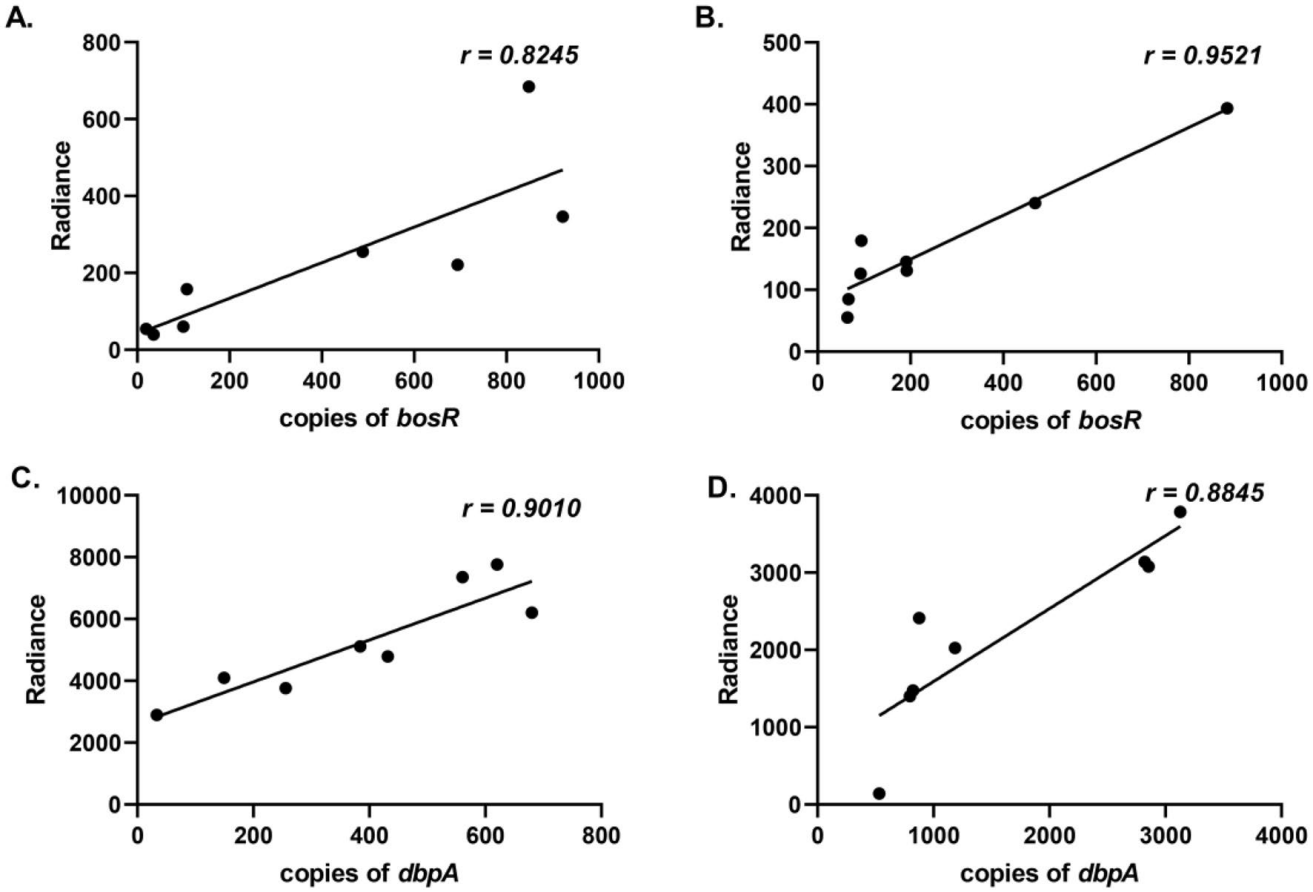
Correlation of reporter driven bioluminescence and native transcript for *bosR* and *dbpBA*. To validate the bioluminescent reporters encoded on a multicopy shuttle vector accurately reflects native transcription a correlation of radiance and copies of the gene of interest within a specific tissue was performed. Total transcript copies of *bosR* from the skin flank (**A**) and heart (**B**) from 10 and 21 dpi was correlated with the representative radiance value. Additionally, *dbpBA* copies from the skin flank (**C**) and heart (**D**) from 10 and 21 dpi was correlated with appropriate radiance.

Data from imaging of P_*bosR*_*-luc* infected tissues was validated by utilizing qRT-PCR to ensure that bioluminescence accurately represents native *bosR* transcript levels. Total copies of *bosR* were quantitated from skin and heart harvested at 10 and 21 dpi (Fig. S2). Radiance from each P_*bosR*_*-luc* tissue from ex vivo imaging samples were correlated with total *bosR* transcript resulting in a strong correlation of 0.8245 and 0.9521 for skin and heart, respectively (Fig. 8A, B). This indicates the bioluminescent emission from P_*bosR*_*-luc* infected tissues is representative of *bosR* transcription during infection.

### Unique expression patterns of *dbpBA* throughout murine infection in all assessed tissues

The expression of *dbpBA* is important to establish a robust colonization during early localized murine infection^39,40,54–56^. Robust expression of *dbpBA* during murine infection suggests a role in dissemination and colonization of specific tissues^52,53^. Our bioluminescent P_*dbp*_*-luc* borrelial strain was used to quantitate gene expression relative to borrelial burden in numerous tissues with the progression of infection (Figs. 6, 7, S1). Bioluminescent emission was readily observed from all tissues at each time points with the exception of joints 4 dpi and hearts at 4 and 7 dpi (Fig. 6). Radiance quantitation clearly demonstrated distinct expression patterns in each tissue that was significantly different by one-way ANOVA for all tissues over time (Fig. S1). Specifically, P_*dbp*_*-luc* in the skin flank peaks on 4 dpi (5.3 × 10^4^ p/s/cm^2^/sr) followed by a dramatic and maintained tenfold reduction at 10dpi out to 21dpi as observed during in vivo imaging (Figs. 6, S1A). The bioluminescent radiance in the lymph node emits steady state levels of *dbpBA* that is substantially higher than P_*bosR*_*-luc* (Fig. S1B). P_*dbp*_*-luc* bioluminescence in the heart increases until reaching a peaking at 1.26 × 10^4^ p/s/cm^2^/sr on 14 dpi (Fig. S1C). Bladder bioluminescence peaks early and rapidly declines similar to P_*dbp*_*-luc* infected skin (Fig. S1D). Joint bioluminescence steadily increases until the final 21 dpi time point with an overall 602-fold increase relative to 4 dpi (Fig. S1E).

Normalization of P_*dbp*_*-luc* radiance values with P_*flaB*_*-luc* reveals unique changes in expression not observed by evaluating P_*dbp*_*-luc* or *dbpBA* alone (Fig. 7). Specifically, skin P_*dbp*_*-luc*/P_*flaB*_*-luc* indicates a higher level of *dbpBA* expression during borrelial colonization at 7 dpi that is significantly different than all compared time points (Fig. 7A). Bioluminescence levels of P_*dbp*_*-luc* are higher than P_*flaB*_*-luc* at 7, 14, and 21 dpi. Permutation analyses of lymph node P_*dbp*_*-luc*/P_*flaB*_*-luc* bioluminescence found no significant differences between time points indicating a steady state expression of *dbpBA* in this tissue unlike the other distal sites evaluated (Fig. 7B). Infected hearts and bladders normalized for borrelial loads demonstrated peak P_*dbp*_*-luc* bioluminescence at 7 dpi and were significantly different from all time points (Fig. 7C, D). Tibiotarsal joints displayed the most distinct bioluminescence profile increasing 31-fold when peaking at 21 dpi (Fig. 7E). As was performed with P_*bosR*_*-luc* infected tissues, qRT-PCR was used to correlate native *dbpBA* transcript levels with bioluminescence from the multicopy reporter (Figs. 8, S2). Correlation values of 0.9010 and 0.8845 for the heart and joint, respectively, were observed at 10 and 21 dpi (Fig. 8C, 8D). This data indicates that bioluminescence quantitated from P_*dbp*_*-luc* encoded on a shuttle vector accurately represents *dbpBA* expression. Overall, *B. burgdorferi dbpBA* is strongly expressed in all tissues at most time points further supporting an important role for this operon during murine infection^27,52,53^. Together, this data demonstrates *dbpBA* regulatory patterns are distinct from expected RpoS driven regulation that was observed previously for P_*ospC*_*-luc*^38^.

## Discussion

Lyme disease progresses in multiple stages from localized infection, through dissemination, and establishes a long-term infection of distal tissues^1–3^. *B. burgdorferi* is a highly invasive bacterial pathogen that responds to distinct environmental pressures as it progresses through stages of mammalian infection^2,57^. Different tissues in the mammalian host presents an unique combination of environmental signals and ligands for interactions requiring *B. burgdorferi* to adapt to each site to colonize and maintain an infection. The borrelial gene regulation that occurs during the different stages of disease and tissues is not fully characterized. Further understanding disseminated or late Lyme disease will provide an opportunity to develop therapeutics beyond the window for effective antibiotic treatment. To date, serodiagnostics tests for Lyme diseases cannot identify early borrelial infections and a human vaccine is not available^58^. Therefore, patients are often diagnosed after dissemination has occurred and symptoms causing severe morbidity have arisen.

To further elucidate the complex spatiotemporal gene regulation of *B. burgdorferi,* we developed bioluminescent borrelial reporter strains allowing us to utilize highly sensitive in vivo imaging to quantitatively evaluate changes in bacterial load and gene expression throughout murine infection and individual tissues^38,39^. In this study, we generated bioluminescent reporter strains for genes encoding a borrelial transcriptional regulator, *bosR*, and adhesive lipoproteins, *dbpBA*, that are known to be important for mammalian infection, in part, due to the interplay with the transcriptional activator RpoS^22–25,31,48^. The absence of *bosR* results in the loss of *rpoS* activation and the reduction of RpoS regulated genes, including *ospC* and *dbpBA*^22–25^. In vivo imaging of P_*bosR*_*-luc* infected mice demonstrated low level expression throughout the 21 day infection relative to constitutively expressed (P_*flaB*_*-luc*) or P_*dbp*_*-luc* infected mice. This was a similar expression pattern to that previously observed with P_*ospC*_*-luc B. burgdorferi* with the exception of notable increase of *ospC* expression at 21 dpi^38^. Individual tissues elicited distinct levels of *bosR* expression with the heart emitting the highest level of bioluminescence, specifically at 4 and 7 dpi. The lowest bioluminescence from P_*bosR*_*-luc* infected tissues were from the inguinal lymph nodes and the bladder. An intermediate level of *bosR* expression was induced in the tibiotarsal joint and skin flanking the inoculation site with both tissues having the highest expression at 4dpi. The tibiotarsal joint also has substantial *bosR* expression at 21 dpi. Together, this data suggest transcriptional regulation of *bosR* occurs during murine infection largely during the early stages of colonizing an individual tissue. While *bosR* is essential for murine infection, low level expression is sufficient to support infection and is not required in all distal tissues. These findings are not in complete agreement with Ouyang et al. that molecularly quantitated *bosR* expression during the tick and murine cycle of infection^53^. Their study found higher levels of *bosR* expression at different time points in some of the individual tissues evaluated in this report. Specifically, *bosR* expression in the skin was abundant and increasing at 7, 14, and 21 dpi. It is not clear the source of the skin sample relative to tick attachment and could contribute to different expression patterns. They also found a substantial increase in *bosR* transcript levels at 7, 14, and 21 dpi in the bladder where in our study we found a relatively steady state low level of expression in this tissue. Differences in findings may be due to routes of infection, sensitivity of detection, and/or distinct methodologies. In vivo imaging allows for less manipulation of the tissues during ex vivo imaging with a high level of sensitivity for viable *B. burgdorferi.* In regards to the Ouyang et al. study, pooling of tissue RNA samples may have also been a contributing factor to distinct findings in regards to *bosR* expression^53^.

BosR, BB0647, is a transcriptional regulator that was initially identified as a Fur-like metalloregulatory transcriptional regulator to combat the oxidative stress response *B. burgdorferi* faces during tick blood meal and mammalian infection^59–62^. More recent studies suggest BosR is a potential global transcriptional regulator for the adaptation to mammalian infection and nutritional requirements^14,63^. As the second member of a three gene operon, *bosR* transcription is driven from two promoters, P_*bb0648*_ and P_*bosR*_, but little is known about the role of the individual promoters on *bosR* expression^33^. Here, we have utilized P_*bosR*_ in our bioluminescent reporter. The transcriptional regulation of *bosR* is similar to other borrelial genes that are responsive to pH, temperature, and growth phase^33,64^. Expression of *bosR* is also induced during nutrient stress in a Rel_Bbu_, responsible for the synthesis and hydrolysis of (p)ppGpp, dependent manner^65^. BadR, OppA4, and CsrA have proposed roles in *bosR* transcriptional regulation^64,66–68^. BadR and OppA4 repress *bosR* expression and the presence of CsrA is responsible for an increase in *bosR* transcription. Ouyang et al. evaluated potential autoregulation of BosR demonstrating minimal reduction in transcription in the absence of *bosR*^33^. Together, this suggests the transcriptional regulation of *bosR* occurs under a variety of conditions. Our findings in this study indicate that *bosR* transcriptional regulation may play a minor role influencing the outcome of borrelial pathogenesis.

It is possible that other levels of regulation or the metal dependent active state of BosR are more important to the regulation of *B. burgdorferi* during mammalian infection. We previously showed that post-transcriptional regulation of BosR occurs dependent on the concentration of CO_2_^14^. Metals have an important role in regulation and active state of BosR. Manganese, zinc, and copper have been shown to post-transcriptionally regulate BosR production^15,17^. Specifically, manganese represses and zinc promotes BosR synthesis^15^. The metal binding ability of BosR is a point of debate in the field. Wang et al. found BosR is able to bind zinc, iron, and copper. In their study, copper and zinc had an inhibitory effect on DNA binding by BosR. A recent study focused on the metal binding ability of the two conserved CXXC motifs within BosR and found these sites were important for zinc binding, but not copper or iron^69^. Mutagenesis of the CXXC motifs reduced the production of RpoS and OspC. A conserved arginine at residue 39 in BosR is important for protein function in regards to combatting oxidative stress, DNA binding, OspC production, and murine infectivity^60,70^. The essential requirement for BosR during mammalian infection may be contributed to the active state of the functional protein through metal binding, oxidative state, and/or DNA binding abilities. The specific mechanisms that post-transcriptionally regulate *bosR* or how they influence pathogenesis in the murine host are not fully characterized. More broadly, the understanding of post-transcriptional regulation and the influence of sRNA encoded with the borrelial genome has only begun to be identified and pursued^71–73^.

We also evaluated the spatiotemporal expression of BosR-Rrp2-RpoN-RpoS regulated genes, *dbpBA*, utilizing in vivo bioluminescence reporter *B. burgdorferi*. During murine infection P_*dbpBA*_*-luc* emits abundant bioluminescence throughout the 21 day infection (Figs. 3, 4). Two peaks are observed during early dissemination (7 dpi) and late infection (21 dpi). This is distinct from two other members of the BosR-Rrp2-RpoN-RpoS pathway, *bosR* and *ospC*. Specifically, the highest P_*dbp*_*-luc* bioluminescence is observed in the skin flank and heart during dissemination (Figs. 6, 7). The *dbpBA* expression is observed in the inguinal lymph node and tibiotarsal joint at a high level for each time point with the highest at late infection. Expression of *ospC* is minimally induced or absent in the inguinal lymph node^38^. Overall, our study demonstrates the overall induction of *dbpBA* expression throughout murine infection in all evaluated tissues. It is also clear that *dbpBA* undergoes RpoS independent regulation during mammalian infection by an unknown mechanism.

*B. burgdorferi* ability to interact with decorin is important for murine infection^41^. Mice lacking decorin are resistant to borrelial infection^74^. Alternatively, *B. burgdorferi* lacking *dbpBA* has an attenuated or a non-infectious phenotype in C3H and Balb/c mice, respectively^39,40^. This correlates with induced expression of *dbpBA* in response to mammalian environmental signals, including low pH, elevated temperature and CO_2_^11,14,48,75^. During tick transmission of *B. burgdorferi,* the expression of *dbpBA* is delayed relative to *ospC* and is not observed prior to dermal colonization^55^. Abundant expression of *dbpBA* throughout murine infection has been observed in other studies^52,53^. The differences in *dbpBA* expression during murine infection and DMC incubation may be due to *B. burgdorferi* ability to interact with host tissues. Specifically, Hodzic et al. observed expression of *dbpBA* in skin, heart, and tibiotarsal joint of C3H and C3H-scid mice over the course of an 8 week infection^52^. The changes in transcript levels reported did not account for fluctuations in bacterial load through the course of infection or between the mouse strains. Higher levels of *dbpBA* expression in C3H-scid were likely due to higher borrelial burden. Additional studies evaluating *dbpBA* expression in SCID mice suggest the humoral response does not influence regulation of these genes in *B. burgdorferi*^76^. Ouyang et al. intradermally infected C3H mice and harvested skin, heart, and bladders for quantitative RT-PCR analysis of borrelial transcriptional response during murine infection normalized to *flaB* transcripts^53^. In this study, *dbpBA* was expressed at high levels in the bladder at all time points out to 50 dpi, whereas skin and heart had much lower or not detectable expression of the transcript. This is distinct from our study where P_*dbp*_-*luc* emits quantitative bioluminescence at all time points. Overall, Ouyang et al. showed lower levels of detection of *dbpBA* at most time points in comparison to our bioluminescent in vivo reporter strain that may be attributed to the methodology requiring more manipulation to isolate RNA and reverse transcriptase-dependent dectection^33^. This suggests that ex vivo imaging of tissues infected with bioluminescent borrelial strains obtains a higher level of sensitivity relative to molecular methods. Our method also has the advantage of evaluating individual tissues and detecting the natural variability that is innate to murine experimental infection.

While our study is insightful to the understanding of borrelial transcriptional regulation during murine infection it is not without limitations. The bioluminescent reporter strains cannot address post-transcriptional or translational regulation that is known to be utilized by *B. burgdorferi*. Nor does it determine the presentation of DbpA on the surface of the bacterial cell or the active state of BosR that requires metal binding and response to the oxidative state of the environment. These considerations require further investigation and methodologies that can be applied during murine infection. A second drawback to this study is the inability to perform tick transmission murine infections because the bioluminescence strains were generated in the ML23 background lacking lp25 that is required for tick and mammalian infection. The presence of *pncA* on the luciferase shuttle vector restores the ability to infect mice and serves as an in vivo selective pressure assuring that bioluminescence will not be lost over time^39,44^. Ideally, a single borrelial strain can be developed with two independent bioluminescent and/or biofluorescent alleles for evaluating bacterial load and expression of a gene of interest in a background strain that can infect ticks and mice while maintaining emission throughout a long term infection.

Future investigation is needed to understand the *B. burgdorferi* genetic regulation necessary for successful dissemination, colonization of distal tissues, and maintenance of long term infection. Specifically, the identification of host-specific environmental signals, the immune response, and host–pathogen interactions that may influence the expression of borrelial genes. Our study, and others, shows that *dbpBA* is distinctly regulated from *ospC* suggesting an unique regulatory mechanism from RpoS occurs during mammalian infection that has not been identified under in vitro cultivation conditions. Regulation of *bosR* is not limited to transcript levels, but includes post-transcription regulation in response to CO_2_ by a yet to be identified mechanism^14^. Recent identification of numerous sRNA encoded in the *B. burgdorferi* genome along with 5′ and 3′ untranslated regions (UTR) are possible candidates to explain the post-transcriptional regulation of *bosR*^73^. We largely do not understand the post-transcriptional regulation that is utilized by *B. burgdorferi* during mammalian infection. By extension, it is highly likely that similar, but distinct regulatory mechanisms are utilized during the tick stage of the pathogenic life cycle.

In this study, we have shown that *B. burgdorferi* uniquely regulates genes dependent upon the tissue that presents unique environmental signals and interactions. This genetic regulatory response varies over time in a manner that may be attributed to changes in the immune response. Specifically, transcriptional regulation of *bosR* and *dbpBA* occurs during murine infection distinctly in various tissues for two genes in the same regulatory pathway. Our study specifically focused on the transcriptional regulation from one of two promoters that drives *bosR* transcription and does not address post-transcriptional regulation of *bosR.* This is the first step in unraveling the complex regulation of *bosR*. The transcriptional regulation of *dbpBA* is likely independent of RpoS during a portion of murine infection. It is unclear when RpoS regulation of *dbpBA* is required. It is likely that other as yet unidentified mammalian specific regulatory mechanism are important for borrelial pathogenesis.

## Materials and methods

### Bacterial strains and culture conditions

Low-passage *B. burgdorferi* B31 were routinely cultured in BSK-II medium supplemented with 6% normal rabbit serum (Pel-Freez, Biologicals, Rogers, AR) (Table 1)^77^. Spirochetes were enumerated using dark-field microscopy. *B. burgdorferi* were grown to mid-exponential phase at 37 °C with in BSK-II medium adjusted to pH 6.8 or pH 7.5 at 1% CO_2_ or pH 7.5 with 1% CO_2_ or 5% CO_2_. All cultures were inoculated at a density of 10^5^ cells/ml and harvested for luminescence and protein samples when cultures reached log phase. When appropriate 300 μg/ml kanamycin was added to the BSK-II medium. *Escherichia coli* were grown in Luria broth (LB) at 37 °C supplemented with the appropriate antibiotics, spectinomycin (100 μg/ml) or kanamycin (50 μg/ml).

### Generated constructs and modification of *B. burgdorferi*

The promoter regions of genes of interest were amplified using forward and reverse primers as listed in Table 2 then cloned into pCR8/GW/TOPO (Invitrogen). Promoter regions for *bosR*, *dbpBA*, and *ospA* were amplified 393 bp, 362 bp, and 431 bp, respectively, upstream of the ATG. A construct encoding Bb*luc* lacking a promoter was generated with SalI, XhoI, ClaI, and NdeI restriction sites for promoter cloning in pCR8/GW/TOPO, designated pJH434. To obtain the final construct, the promoter linked to Bb*luc* was cloned into pBBE22^44^. The final construct was screened by restriction digest, luminescence, and confirmed by sequence analysis. This cloning design resulted in borrelial luciferase reporters for P_*bosR*_-*luc* (pJH481), P_*dbp*_-*luc* (pJH488), and P_*ospA*_-*luc* (pJH486). The transformations of *B. burgdorferi* ML23 with bioluminescent reporter shuttle vectors were carried out as described previously^78^. Transformed cultures were plated by limiting dilution in the presence of antibiotic selection. Positive transformants for were confirmed by bioluminescence assays, PCR screening for reporter plasmid and borrelial plasmid content^45^. For clarity the resulting strains ML23 pJH481, ML23 pJH488, and ML23 pJH482 are designated P_*bosR*_*-luc*, P_*dbp*_*-luc*, and P_*ospA*_*-luc*, respectively, (Table 1). Previously generated ML23 pBBE22*luc* is also designated P_*flaB*_*-luc*^39^.

**Table 2:** Primers used in this study.

Primer	Sequence (5′ to 3′)^a^
PbosR F-SalI	ACGCGTCGACCTAAAGGAAATGATAAAAACAGC
PbosR R-NdeI	ACGCCATATGTATGATTATACCTTTTTTG
Pdbp F-SalI	ACGCGTCGACCTCTTTTATTTTTAAGACC
Pdbp R-NdeI	ACGCCATATGTTTTTCCTCCTTCTATTAA
PospA F-SalI	ACGCGTCGACCATTTCTTGTGAAGAC
PospA R-NdeI	ACGCCATATGAATATATTCTCCTTTTA
Bbluc F MCS	ACGCGTCGACACGCCTCGAGATCGATACGCCATATGGAGGATGCCAAAAACATT
Bbluc R BamHI	ACGCGGATCCAAGCTTTTATTATACA
RT-Bactin F	ACGCAGAGGGAAATCGTGCGTGAC
RT-Bactin R	ACGCGGGAGGAAGAGGATGCGGCAG
RT-recAB F	GTGGATCTATTGTATTAGATGAGGCT
RT-recAB R	GCCAAAGTTCTGCAACATTAACACCT
dbpA std F	GTTAACCTACTTATATCATGTGGACT
dbpA std R	GATGGATTTGGTTGGGTATTGT
bosR std F	ATGAACGACAACATAATAGACG
bosR std R	TAAAGTGATTTCCTTGTTCTC
dbpA qRT-PCR F	CAGATGCAGCTGAAGAGAATCCT
dbpA qRT-PCR R	ACCCTTTGTAATTTTTCTCTCATTTTT
bosR qRT-PCR F	ACCCTATTCAACTTGACGATATTAAAGAT
bosR qRT-PCR R	GCCCTGAGTAAATGATTTCAATAGATT

^a^The underlined sequences are restriction enzyme sites.

### SDS-PAGE and immunoblot analysis

Sodium dodecyl sulfate–polyacrylamide gel electrophoresis (SDS-PAGE) and immunoblot analysis were performed as previously described^9^. Briefly, cell lysates were subjected to SDS-PAGE and transferred to a PVDF membrane for Western analysis. Proteins were detected using mouse monoclonal antisera to *B. burgdorferi* flagellum (Affinity BioReagents), rabbit anti-DbpA, rabbit anti-BosR, mouse anti-OspA, respectively. The following secondary antibodies were used for additional amplification: rabbit anti-mouse IgG conjugated with horseradish peroxidase (HRP), and anti-rabbit HRP.

### In vitro bioluminescence assays

Three independent cultures of P_*flaB*_*-luc*, P_*bosR*_*-luc,* P_*dbp*_*-luc* and P_*ospA*_*-luc* were grown to mid-log phase and concentrated to 10^7^ cells/ml. Cells were serially diluted from 10^6^ to 10 cells and 100 μl of each appropriate dilution were distributed in a white flat-bottom microtiter 96 well-plate. Each sample for each strain was treated with a fresh 2 mM D-luciferin (GoldBio) diluted in PBS and luminescence was measured using 2104 EnVision Multilabel Reader (Perkin Elmer). The values of three independent cultures were averaged and the standard error was calculated.

### In vivo and ex vivo bioluminescence infectivity studies and quantitation

Mice were cared for under standard ABSL-2 parameters during housing and IVIS imaging under the supervision of a Texas A & M University veterinarian. General health and wellness of the mice were monitored daily. No unexpected illnesses, distress, or deaths occurred during the time course of the infection. During imaging mice were given isoflurane as an anesthetic. Per the guidelines of the American Veterinary Medical Association (AVMA) and as approved by the Texas A & M University Institutional Animal Care and Use Committee (IACUC) mice were euthanized at the predetermined time points.

Bioluminescent imaging of mice and tissues harvest was performed as previously described^38,39^. Groups of 5 female 6–8 week old Balb/c mice (Charles Rivers) were infected by intraperitoneal injection (IP) with 10^5^ *B. burgdorferi* P_*flaB*_*-luc,* P_*bosR*_*-luc,* P_*dbp*_*-luc,* or P_*ospA*_*-luc*. 5 mg D-luciferin dissolved in PBS was delivered to 4 of 5 mice by intraperitoneal (IP) injection. After 10 min mice were imaged at 2 h and 1, 4, 7, 10, 14 and 21 days post-infection (dpi) using the Perkin Elmer IVIS Spectrum live imaging system. One mouse not treated with D-luciferin serves as a background control for each group and time point. Exposures between 600–60,000 counts were used to quantitate bioluminescence, photons/sec (p/s) or radiance (p/s/cm^2^/sr) utilizing the regions of interest (ROI) tool from the Perkin Elmer Living Image Software. Bioluminescence from D-luciferin treated mice were normalized by subtracting background bioluminescence from the untreated mouse. Normalized bioluminescence values were averaged and standard error calculated. All images are a 10 min exposure and normalized for background across all borrelial strains and time points as represented by the colorimetric radiance scale.

Bioluminescence of individual infected tissues were measured during ex vivo imaging after mice were treated with 10 mg of D-luciferin 10 min prior to sacrifice and harvesting of skin, inguinal lymph node, heart, bladder, and tibiotarsal joint. Harvested tissues were transferred to 4 mM D-luciferin and 2 mM ATP solution and soaked for 3 min to avoid dehydration during imaging. As with in vivo imaging, one mouse was not treated with D-luciferin and tissues were transferred to 1X PBS prior to imaging. Bioluminescence of tissues was performed as described above for in vivo imaging. Tissues were flash frozen in liquid nitrogen and stored at -80 °C until isolation of total RNA for qRT-PCR. Skin samples were also collected to determine bacterial load.

### Quantitative PCR and RT-PCR analysis

As previously performed, DNA or RNA was extracted from infected tissues to quantitate borrelial burden or native transcript levels^38^. *B. burgdorferi* infected skin flanks were processed with the Roche High Pure PCR Template Preparation kit to isolate genomic DNA. The Applied Biosystems ABI Step One was used to quantitate bacterial equivalents from infected mouse tissues. Borrelial genomic equivalents were quantitated in numbers of borrelial *recA* per 10^5^ mouse *β-actin* as previously described from 100 ng of genomic DNA (Table 2)^40^. A C_t_ standard curve of calculated amounts of pβ-actin and p*recA* standard plasmids to determine quantities from the C_t_ values of the experimental samples. Samples were measured in technical triplicate.

RNA was extracted from infected tissue by phase separation with Trizol per manufacture instructions (ThermoFisher) as previously performed^38^. Briefly, tissues were homogenized in Trizol, then treated with 200 μl of chloroform per ml of Trizol. Centrifugation of samples allowed phase separation and the upper aqueous phase containing RNA was precipitated. Total RNA (≤ 30 μg) was treated with 5 units Roche recombinant DNase to remove contaminating DNA from the sample. cDNA was generated from 3 μg of DNase treated RNA using Superscript III and random hexamers in a 20 μl reaction per manufacturer instructions (ThermoFisher). Transcript levels for each target, *bosR* and *dbpA,* was evaluated by qPCR with 3 μl of cDNA, 360 nM primers, and PowerUp Sybr (Thermofisher) (Table 2). A standard curve was utilized to determine total transcript levels of each tissue evaluated.

### Statistical analyses

Statistical analysis of in vitro luminescence assays, in vivo bioluminescence (p/s), and ex vivo bioluminescence (p/s/cm^2^/sr) was performed using GraphPad Prism (GraphPad Software, Inc, La Jolla, CA). Significance was determined by *p* values equal to or less than 0.05. Bioluminescence temporal differences of individual strains was analyzed by one-way analysis of variance (ANOVA). Mann–Whitney one-tail test compared differences between bacterial strains at a single time point. Correlation was performed between quantitated bioluminescence and native transcript levels. To determine the significant differences over time of P_*bosR*_*-luc*/P_*flaB*_*-luc* and P_*dbp*_*-luc*/P_*flaB*_*-luc* radiance ratios we used permutation tests to compute sampling distributions as previously described^38^. Permutation tests were performed using R, a freely available language and environment for statistical computing and graphics (ver. 3.2.3; https://cran.r-project.org/).

### Ethics statement

In accordance with National Institute of Health (NIH) Guide for Care and Use of Laboratory Animals and Association for Assessment and Accreditation of Laboratory Animal Care (AAALAC) guidelines animal experiments were performed as described above. The Texas A & M University Institutional Animal Care and Use Committee (IACUC) approved all animal protocols and procedures, including but not limited to method for euthanasian.

## Supplementary information

Supplementary information.

Supplementary Legends

Supplementary Figures.
