# Supplementary material for: Borrelia burgdorferi spatiotemporal regulation of transcriptional regulator bosR and decorin binding protein during murine infection
Source: Sci Rep. 2020 Jul 27;10:12534. doi: 10.1038/s41598-020-69212-7 (PMC7385660; doi:10.1038/s41598-020-69212-7)
Supplement: Supplementary file 2 — Supplementary Legends [file 41598_2020_69212_MOESM2_ESM.docx]

**Supplemental Figure 1.** **Radiance of individual murine tissues infected with bioluminescent *B. burgdorferi*.** *Ex vivo* bioluminescence exposures of individual murine tissues were between 600-60,000 counts for the accurate quantitation of radiance (p/s/cm^2^/sr). Tissues from 4 mice normalized for background were averaged and standard error represented by error bars. Two-way ANOVA analysis of radiance indicated the (A) underside skin (P*_flaB_-luc p<* 0.0001, P*_bosR_-luc p* < 0.0028 , P*_dbp_-luc* *p* < 0.0001), (B) lymph node (P*_flaB_-luc p<* 0.0588, P*_bosR_-luc p* < 0.0740, P*_dbp_-luc* *p* < 0.0072), (C) heart (P*_flaB_-luc p<* 0.0168, P*_bosR_-luc p* < 0.0035, P*_dbp_-luc* *p* < 0.0001), (D) bladder (P*_flaB_-luc p<* 0.0696, P*_bosR_-luc p* < 0.0155, P*_dbp_-luc* *p* < 0.0497), and (E) joint (P*_flaB_-luc p<* 0.0007, P*_bosR_-luc p* < 0.2140, P*_dbp_-luc* *p* < 0.4817) were significantly different between strains and over time points for skin and heart.

**Supplemental Figure 2. Analysis of native *bosR* and *dbpBA* transcripts from tissues infected bioluminescent *B. burgdorferi* reporter strains.** (A) Skin and (B) heart tissues harvested from P*_bosR_-luc* infected mice at 10 and 21 dpi were utilized to isolate total RNA and converted to cDNA. Native *bosR* transcripts were assessed by qPCR. Total RNA was extracted from *B. burgdorferi* P*_dbp_-luc* infected (C) heart and (D) joints to quantitate native *dbpBA* transcripts at 10 and 21 dpi.

**Supplemental Figure 3. Western blots of *in vitro* grown reporter strains.** Cell lysates from (A) P*_dbp_-luc*, (B) P*_ospA_-luc*, (C & D) P*_bosR_-luc* and P*_flaB_-luc* were immunoblotted and probed with anti-sera to antigen indicated on the left. Constitutively produced borrelial FlaB was used as a control for cell equivalents between samples. Cultures were grown in triplicate under different pH or CO_2_ levels. Uncropped Western blot showing the levels of DbpA, OspA, and BosR under each condition.
