# Supplementary material for: Borrelia burgdorferi spatiotemporal regulation of transcriptional regulator bosR and decorin binding protein during murine infection
Source: Sci Rep. 2020 Jul 27;10:12534. doi: 10.1038/s41598-020-69212-7 (PMC7385660; doi:10.1038/s41598-020-69212-7)

Borrelia burgdorferi spatiotemporal regulation of transcriptional regulator *bosR* and decorin binding protein during murine infection

Running title: *Borrelia burgdorferi* gene regulation during mammalian infection

Elizabeth P. Saputra[1], Jerome P. Trzeciakowski[2], and Jenny A. Hyde[1*]

[1]Department of Microbial Pathogenesis and Immunology and [2] Department of Medical Physiology, College of Medicine, Texas A&M Health Science Center, Bryan, Texas, United States of America.

Key words: Borrelia burgdorferi, Lyme disease, bioluminescence, *in vivo* imaging, infection, bosR, dbpBA, decorin, gene regulation, ospA, ospC

* corresponding author current email: jhyde@tamu.edu

Supplemental Figure 1

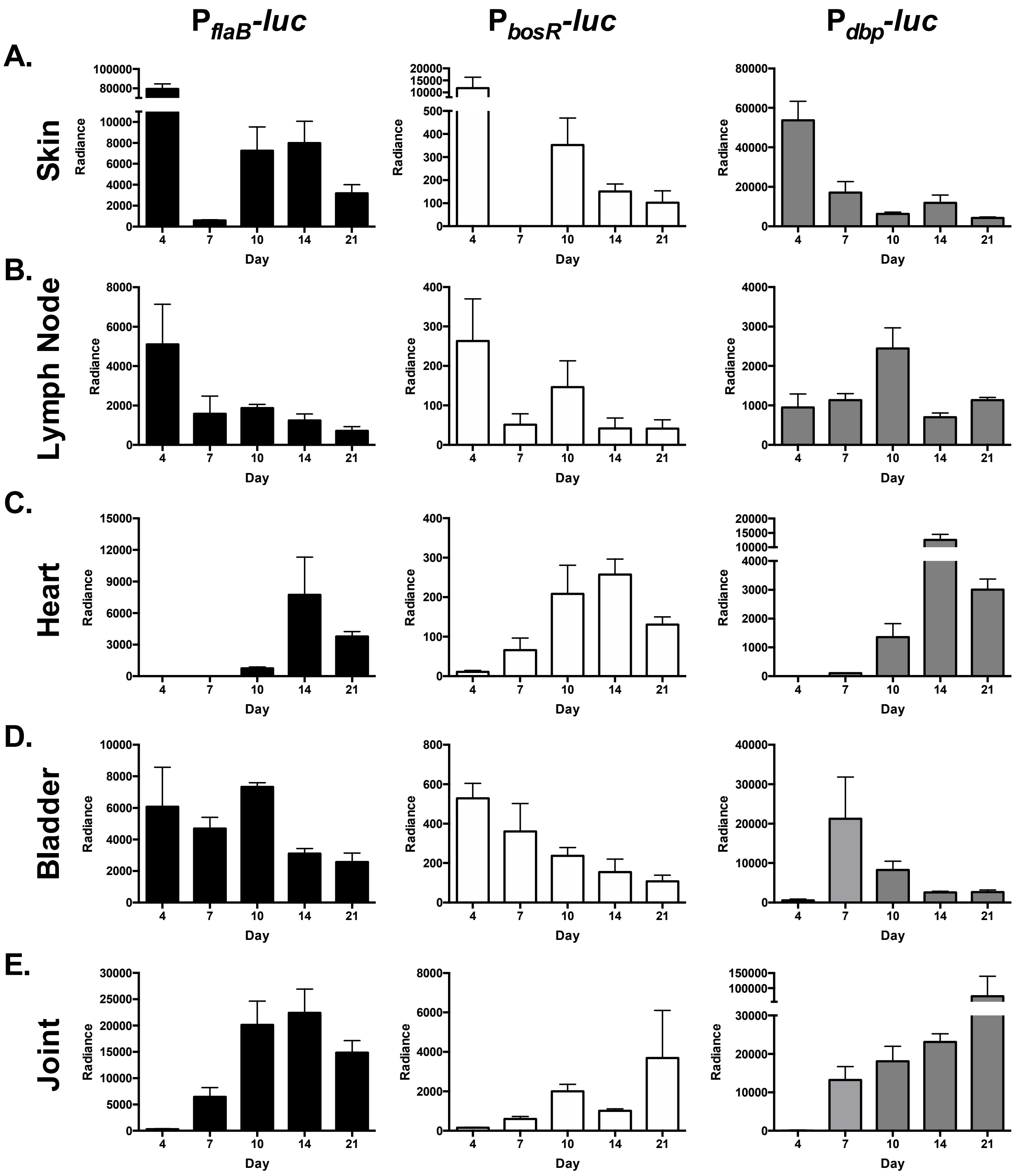

Supplemental Figure 2

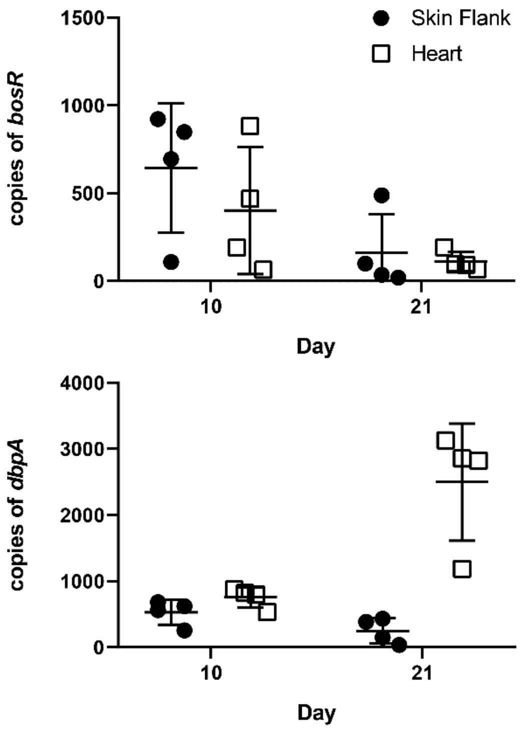

Supplemental Figure 3

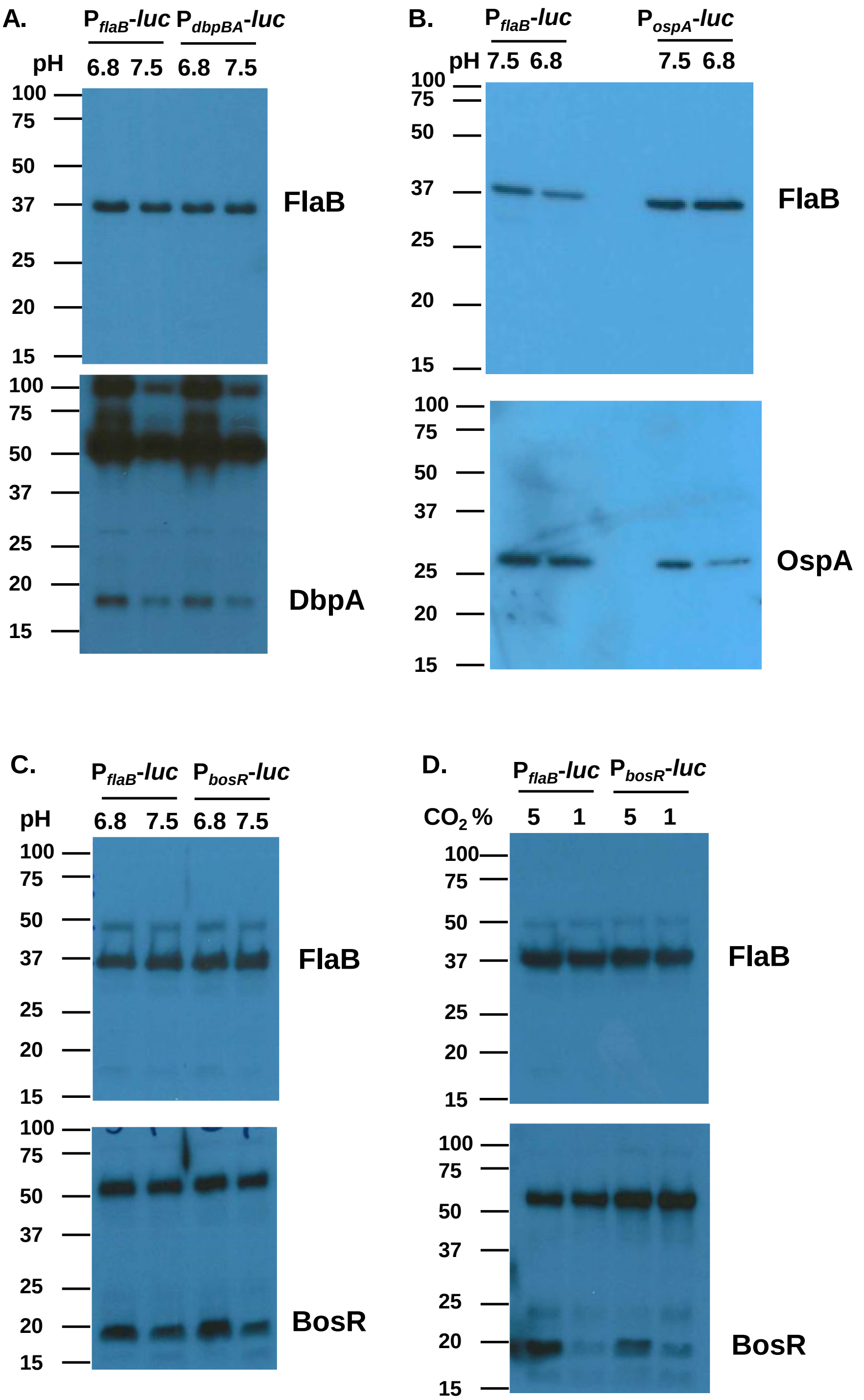

Supplement: Supplementary file 3 — Supplementary Figures. [file 41598_2020_69212_MOESM3_ESM.pdf]
